# Letter to the Editor Regarding “Efficacy and safety of adjunctive corticosteroids in the treatment of severe community-acquired pneumonia: a systematic review and meta-analysis of randomized controlled trials”

**DOI:** 10.1186/s13054-023-04576-6

**Published:** 2023-07-24

**Authors:** Shengfeng Wang, Jian Liu, Lingtong Huang

**Affiliations:** 1 Department of Critical Care Units, The Second Affiliated Hospital of Zhengzhou University School of Medicine, Zhengzhou, China; 2 Department of Critical Care Units, The First Affiliated Hospital, Zhejiang University School of Medicine, Hangzhou, China

Dear Editor,

We read the article by Wu et al. published on Critical Care with great interest [1]. In light of the recent disclosure of clinical trials regarding the use of corticosteroids in the treatment of severe community-acquired pneumonia (CAP), it becomes necessary to provide an updated meta-analysis in this field [2]. Based on our ongoing meta-analysis investigating the role of corticosteroids in CAP (PROSPERO register number: CRD42023415719), we believe that there are still certain issues that need to be clarified in the study conducted by Wu et al.

Firstly, the study population of this research focused on severe CAP and did not include patients with COVID-19. However, the authors did not explicitly state in the article or the registered protocol that COVID-19 patients were excluded from this study. Undoubtedly, the inclusion of clinical trials involving COVID-19 would not alter the fact that patients with severe CAP would benefit from corticosteroid treatment. It is still necessary for the authors to make a note of this point in their article which is a point explicitly mentioned in our forthcoming meta-analysis (PROSPERO register number: CRD42023415719).

Secondly, there may be a need for some data to be revised. Certain key studies were not included in the analysis, such as the study by Snijders et al., which also involved a significant proportion of severe CAP [3]. Additionally, the study by Meduri et al. did not provide a 30-day mortality rate; instead, they provided in-hospital mortality and 60-day mortality [4]. Similarly, the study by Confalonier et al. only provided in-hospital mortality rates [5]. These cannot simply be classified as 30-day mortality.

Lastly, the approved registration date of the authors' study in PROSPERO (PROSPERO register number: CRD42023421345; day of registration: 06 May 2023) appears to be after the submission to Critical Care. This discrepancy should also be acknowledged in the article. As we conducted a search in PROSPERO during our own registration process and did not find any similar meta-analyses registered before, we have concerns about the early publication of this manuscript.

There is no doubt that this study deserves publication, and we congratulate the authors for a detailed meta-analysis. At the same time, most of the conclusions of Wu et al.'s study are similar to ours, which adds credibility to this study. However, the above problems still need to be declared in the article.

## Data Availability

None.
